# Recovery response comparisons between variable resistance and long and short muscle length isometric exercise

**DOI:** 10.1007/s00421-025-05958-0

**Published:** 2025-09-13

**Authors:** Giuseppe Rosaci, Franco Merni, Samuele Marcora, Sandro Bartolomei

**Affiliations:** Department for Life Quality Studies, University of Bologna, Bologna, 40126 Italy

**Keywords:** Elastic bands, Image texture, Muscle architecture, Muscle damage, Strength

## Abstract

Isometric exercises at long muscle length (LML) and short muscle length (SML), and variable resistance (VAR) exercises, are effective to achieve neuromuscular and morphological adaptation. To date, no studies have compared pectoralis major muscle recovery after these modalities. Therefore, this study aimed to compare the muscle damage and recovery after LML, SML, and VAR in trained men. Twelve participants (age: 25 ± 4 y, height: 178 ± 7 cm, body weight: 82 ± 10 kg, training experience: 7 ± 4 y) completed the protocols in a random order with a 10-day washout period. Assessments occurred pre-exercise (BL) and at 15 min (P-15 min), 24 h (P-24 h), and 48 h (P-48 h) post-exercise, evaluating muscle thickness (MT), echo intensity (EI), isometric peak force, average power at bench press throw power test (BPT), and muscle soreness. Blood samples were also collected at BL and at P-24 h, and creatine phosphokinase (CPK) was measured. Changes in MT at P-15 min and P-24 h were more elevated following VAR compared to SML and LML (*p* = 0.003; η^2^_p_ = 0.271). No condition × time interactions were found for EI (*p* = 0.233), peak force (*p* > 0.319), BPT (*p* = 0.614), and muscle soreness (*p* = 0.115). The EI, peak force, and BPT parameters returned to baseline at P-24 h, while muscle soreness persisted for 48 h without any significant differences between protocols. All exercise protocols resulted in similar elevations of CPK (*p* = 0.727; 387 ± 159, 396 ± 199 and 362 ± 170 U/L for LML, SML and VAR, respectively). In conclusion, all exercise protocols cause muscle damage. However, the mechanical and metabolic stress of VAR may prolong the recovery of initial muscle architecture compared to LML and SML.

## Introduction

Isometric training at long muscle length (LML), isometric training at short muscle length (SML), and training with variable resistance (VAR) across the full range of motion represent effective methods to achieve the desired neuromuscular adaptations that may improve sport-specific performance tasks (Oranchuk et al. 2019; Rivière et al. 2017). Isometric exercise performed against an immovable object at LML (defined as pushing/pulling isometric muscle actions) (Oranchuk et al. 2024) shows greater effects on muscle hypertrophy (Alegre et al. 2014), fascicle length (Akagi et al. 2020), tendon stiffness (Kubo et al. 2006) and peak force compared to isometric exercise at SML (Bandy and Hanten 1993; Kubo et al. 2006; Alegre et al. 2014). These differences may be related to a higher muscle excitation (Marchetti et al. 2016), internal force (Kubo et al. 2006), muscle oxygen consumption (de Ruiter et al. 2005; Kooistra et al. 2008), and sarcomeres overstretching (Philippou et al. 2004) that characterize isometric exercises performed at LML compared to SML. VAR training, which provides extra load in some portions of the range of motion, may theoretically optimize the load in some exercises (e.g., squat, bench press) (Stevenson et al. 2010; Saeterbakken et al. 2016), and has been associated with larger improvements in strength (Anderson et al. 2008), velocity, and power compared to traditional resistance training (Rivière et al. 2017). These larger neuromuscular improvements observed in athletes using VAR training, compared to traditional resistance training, may be related to an increased muscle tension in the final portion of the concentric phase and in the first portion of the eccentric phase of the movement (Anderson et al. 2008), leading to a higher electromyographic activity (Cronin et al. 2003).

Although chronic adaptations to both isometric (Oranchuk et al. 2019) and VAR resistance training are well documented (Soria-Gila et al. 2015), the acute effects on the recovery response remain poorly understood. Most of the studies investigating recovery following isometric exercise at different muscle lengths were focused on the leg extensor (McMahon and Onambele-Pearson 2024) and elbow flexor muscles (Jones et al. 1989; Philippou et al. 2003; Allen et al. 2018). These studies reported greater impairments in neuromuscular performance and higher levels of muscle damage and soreness, following isometric exercise at LML compared to SML. Curiously, greater reductions in peak force of the quadriceps muscles following the LML isometric protocol compared to the SML were not associated with greater changes in muscle architecture (McMahon and Onambele-Pearson 2024). Regarding VAR exercise protocols, Walker and colleagues (Walker et al. 2013) investigated recovery using a cam-based resistance system and found larger declines in performance and higher fatigue levels in the knee extensors compared to traditional resistance exercise (Walker et al. 2013). To date, no studies have examined fatigue or recovery responses following isometric LML, isometric SML, and VAR exercise in upper body muscles.

Thus, the aim of the present study was to compare the acute effects and the recovery responses following three exercise protocols involving the pectoralis major muscles in resistance trained men: isometric exercise performed at LML, isometric exercise at SML, and VAR elastic band-resisted exercise. The authors hypothesized that longer recovery responses may characterize VAR elastic band-resisted exercise compared to isometric exercise at both LML and SML. The authors also hypothesized that more muscle damage may occur following isometric exercise at LML and VAR elastic band-resisted exercise compared to isometric exercise at SML.

## Methods

### Study design

**Fig. 1:**
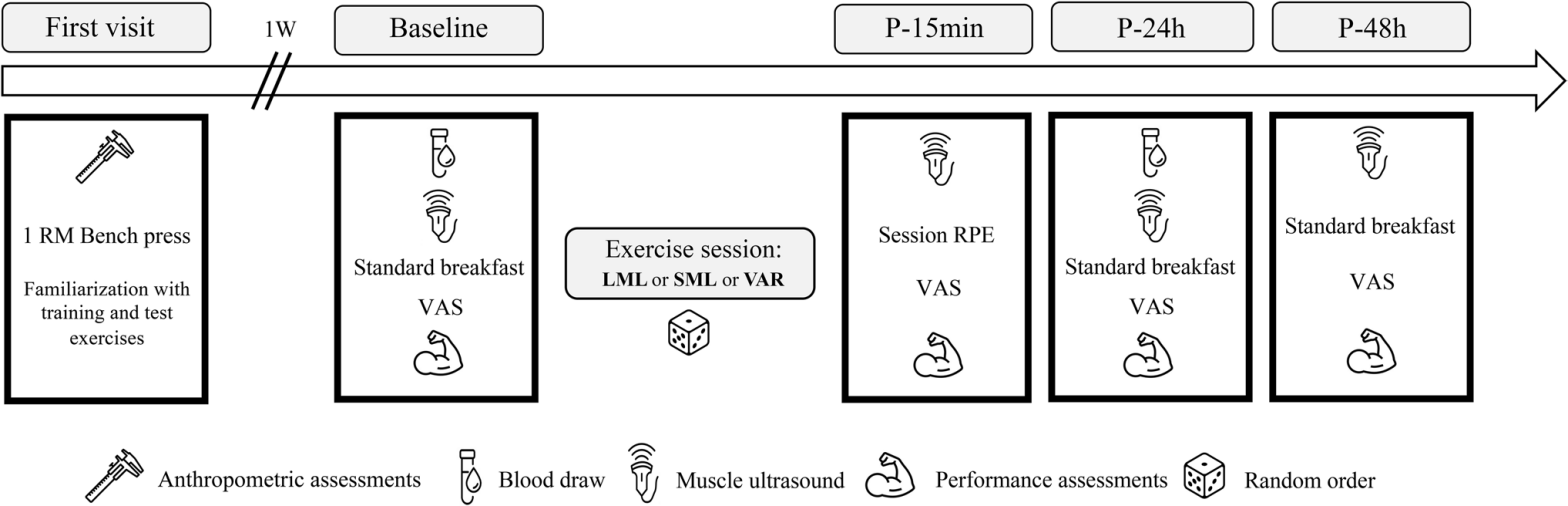
Experimental design of the study. All the exercise sessions mainly targeted the pectoralis major muscles. *1-RM* one repetition maximum, *VAS* visual analog scale, *LML* long muscle length, *SML* short muscle length, *VAR* variable resistance, *P-15 min* 15 min post-exercise, *P-24 h* 24 h post-exercise, *P-48 h* 48 h post-exercise

The experimental design followed a randomized, counterbalanced crossover approach, in which all the participants performed the three exercise protocols (LML, SML and VAR), in randomized order. In the first visit, maximum upper body strength and anthropometric measurements were assessed. In the same visit, participants were familiarized with the isometric and variable resistance exercises included in the experimental study. Each participant returned to the laboratory at least 4 days following their initial visit, to perform either the LML, SML, or the VAR protocol. Prior to (baseline, BL) and 15 min (P-15 min), 24 h (P-24 h), and 48 h (P-48 h) post each exercise protocol, they were tested for muscle architecture, performance and muscle soreness. Blood samples were taken at BL and P-24 h. After a washout period of at least 10 days, participants reported back to the laboratory and completed another exercise protocol (Fig. 1).

### Participants

A convenience sample of 12 resistance trained men (age: 25.3 ± 4.11 years; height: 177.6 ± 7.3 cm; body mass: 81.6 ± 10.1 kg; training experience: 6.7 ± 3.7 years; bench press 1-RM: 108.5 ± 21.6 kg) volunteered to participate in this study. Inclusion criteria required participants to be between the age of 18 and 35 years, with a minimum of 2 years of resistance training experience. In addition, participants were required to be able to bench press at least their body weight, to be healthy and free from musculoskeletal injuries in the year prior to the study. All participants read and signed an informed consent, and the experiment was approved by the local bioethics committee (n. 0070230, 29 February 2024). Participants were asked to abstain from physical activity for 48 h prior to the beginning of each protocol and were not allowed to drink alcohol or coffee in the test days. In addition, participants were instructed not to eat or drink (except water) within 10 h of reporting to the laboratory for each experimental trial.

### Exercises protocols

**Fig. 2:**
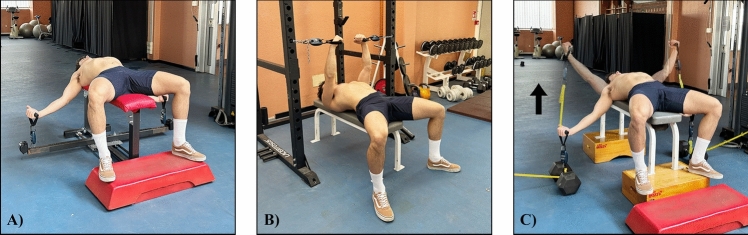
Exercise settings for long muscle length (LML) (**A**), short muscle length (SML) (**B**), and variable resistance (VAR) exercise (**C**)

Both isometric exercise protocols consisted of six sets of five pushing isometric muscle actions (PIMAs) of 5 s performed at LML or SML, with 5 s of rest between the contractions and 75 s of between-sets rest. All the isometric contractions were performed with maximum effort (maximum voluntary isometric contractions). In the VAR exercise protocol, the resistance was set in relation to the previously measured individual peak force registered at SML and LML test. Specifically, the elastic bands were calibrated to obtain a resistance corresponding to 90% of the peak force registered in SML, at the end of the concentric phase, and a force corresponding to 20% of the peak force registered at LML, at the end of the eccentric phase. Each repetition of VAR consisted of a 2.5 s concentric and a 2.5 s eccentric phase. The duration of both phases was monitored by a digital metronome. A between-repetitions rest of 5 s was observed also during VAR. Thus, the total time under tension and the recovery time between sets and repetitions, were equated in LML, SML, and VAR. During the VAR exercise protocol, the resistance bands were perpendicular to the ground. Two operators adjusted the resistance of the bands if the participants were unable to complete the repetitions provided. The movement started from maximum shoulder abduction (about −45°, similar to the LML isometric test), and continued until the arm formed a 45°angle with the horizontal plane (total range of motion: from −45° and to + 45°). The force produced in each isometric contraction of LML, SML, and in each repetition of VAR, was monitored using a wireless dynamometer (K-Pull, Kinvent Physio, Montpellier, 3400, France). Then, peak forces registered in each contraction/repetition of each set were averaged and the percentage decrease in this parameter between the first and the last set of each protocol was calculated as the fatigue index. The settings of the different exercise protocols are reported in Fig. 2.

### Strength and power testing

A standardized warm-up consisting of 5 min on a cycle ergometer against a light resistance, ten body weight squats, ten body weight walking lunges, ten dynamic walking hamstring stretches, ten dynamic walking quadriceps stretches, and five push-ups was performed prior to each testing session at BL, P-24 h,and P-48 h (Bartolomei et al. 2023). The one repetition maximum (1RM) bench press was assessed as previously described by Hoffman (Hoffman 2014) at least 4 days prior to performing the first exercise protocol. To determine their 1RM, three to four attempts were performed, with a rest period of 3–5 min between each attempt. Trials not meeting the range of motion criteria for each exercise or where technique was not appropriate were discarded. Arm length was measured from the superolateral aspect of the acromion to the most distal palpable point of the styloid process of the radius (Norton 2018). A bench press throw power test (BPT), performed at 30% of the previously tested 1RM, was performed at BL, P-15 min, P-24 h, and P-48 h. Participants were instructed to push as explosively as possible until full arm extension and to project the bar as high as possible. Two spotters were placed at each side of the Smith machine to decelerate the bar during the descending phase. Two sets of two repetitions were required with a recovery time of 20 s between each repetition and a 3 min recovery time was used between each set (Bartolomei et al. 2018). An optical encoder (Tendo Unit model V104; Tendo Sports Machines, Trencin, Slovak Republic) was used to measure the average power during all repetitions. The intraclass correlation coefficient (ICC) for the BPT was 0.97 (SEM: 13.1 W). At the same time points (BL, P-15 min, P-24 h and P-48 h), participants performed a maximum isometric force test for the pectoralis major muscles, at LML SML, in randomized order. LML assessments required an apparatus consisting of a horizontal bench provided by two extendable arms positioned at the floor level (Fig. 2A). A wireless dynamometer (Kinvent Physio, Montpellier, 3400, France) was attached to the end of each extendable arm and sampled at 1000 Hz. This apparatus allowed to measure the peak force and to adjust the settings based on the different anthropometric characteristics of the participants (e.g., arm length). The LML isometric test was performed in a shoulder abduction position (about—45°). The isometric test at SML was performed on a power rack (Fig. 2B), with the participants arms parallel and at 90° to the ground. During all the assessments and exercise protocols, the elbow was maintained at 10° of flexion and that angle was monitored using a digital goniometer (K-Move, Kinvent Physio, Montpellier, 3400, France). During the isometric assessments, participants were asked to pull as hard and as fast as possible (Haff et al. 2015) and were verbally encouraged. The same qualified investigators supervised the testing sessions and the exercise protocols. The ICCs were 0.90 (SEM: 14.20N) and 0.94 (SEM: 11.0N) for LML peak force and SML peak force, respectively. The internal force in both LML and SML tests was estimated by dividing the external torque by the moment arm of the pectoralis major. External torque was calculated as the product of the force measured by the dynamometer, the lever arm length, and the sine of the angle between the force vector and the body segment (Robertson et al. 2013). This torque was then divided by the specific moment arm (0.0450 m) of the pectoralis major during the horizontal flexion at 90° of humeral elevation (Kuechle et al. 1997) assuming the pectoralis major muscle as the sole contributor of the internal force.

### Blood sample

Blood samples were collected prior to and 24 h post (P-24 h) each exercise protocol, from a superficial forearm vein using a single-use disposable needle. Participants were positioned in a supine posture for at least 15 min before sampling to ensure accurate measurements (Lippi et al. 2015). After the baseline blood samples, participants ate a standardized breakfast consisting of two regular cereal bars (Coco Pops^®^, Kellogg’s, USA; 1.1 g of protein, 14.0 g of carbohydrates, and 2.2 g of fat). The blood samples were stored away from light at 2–4° for no more than 48 h. All blood samples were collected into two Vacutainer^®^ tubes, containing anticlotting agent (6 mL). The concentration of creatine phosphokinase (CPK) in serum was assessed using a kinetic method optimized according to DGKC-IFCC (Deutsche Gesellschaft für Klinische Chemie—International Federation of Clinical Chemistry and Laboratory Medicine) guidelines with SGM Italia reagent kit. The coefficient of variation for CPK was 3.25%.

### Dietary logs

The participants were asked to record as accurately as possible everything they ate and drank during each 3-day trial. They were also requested to duplicate the content, quantity, and timing of their daily diet during the 24 h prior. The USDA Nutritional Database (US Department of Agriculture, Beltsville, MD, USA) was used to analyze total calories, carbohydrates, protein, and fat.

### Ultrasound measurements

Non-invasive skeletal muscle ultrasound images were collected from the participant’s right side, prior to the warm-up. The pectoralis major muscle thickness (MT) was measured at the site between the third and fourth costa below the center of the clavicle (Nigro et al. 2024). Muscle thickness was measured with a single perpendicular line from the superficial aponeurosis to the deep aponeurosis. The thickness of subcutaneous fat was quantified as the distance between the skin–muscle interface and the superior border of the muscle’s aponeurosis (Ryan et al. 2016). Three consecutive images were acquired and analyzed by the same technician. A 12-MHz linear probe (Echo Wave 2, Telemed Ultrasound Medical System, Milan, Italy), coated with water-soluble transmission gel to optimize spatial resolution, was used to acquire all ultrasound images. The probe was positioned on the skin surface without depressing the dermal layer, and the view mode (gain = 125 dB; image depth = 5 cm) was used to obtain image from PEC. Measurements were taken with the participant in the supine decubitus position for PEC measurements. Measurement required participants to lie on the examination table for at least 15 min before images were taken. During the measurements, participants were asked to relax their arm and chest muscles and to maintain the supine decubitus position. The average of the three MT measurements was used for statistical analysis. The intraclass correlation coefficient was 0.95 (SEM = 1.05 mm). The images were analyzed using a specific software (ImageJ; National Institutes of Health, USA) to calculate the echo intensity (EI) and five parameters of muscle texture (Angular Second Moment, Contrast, Correlation, Entropy, and Inverse Difference Moment). Muscle texture features were analyzed using the GLCM Texture Plugin (version 0.4) (Oranchuk et al. 2025), with a pixel distance setting of 1 and a directional angle of 180°. For each image, the largest possible region of interest was manually selected using the polygon selection tool (Lateef et al. 2024) and consistently applied across analyses. In addition, the raw EI was corrected for subcutaneous fat using the following equation (Young et al. 2015): $Raw EI+(subcutaneous fat* 40.5278)$.

### Muscle soreness

A 100-mm visual analog scale (VAS) (Lee et al. 1991; Bijur et al. 2001) was used to assess the participants muscle soreness of the pectoralis major muscles. No soreness was recorded as 0 and the worst possible soreness or pain as 100. Soreness intensity was evaluated at BL, P-30 min, P-24 h, P-48 h.

### Statistical analysis

A Shapiro–Wilk test was used to test the normal distribution of the data. If the assumption of sphericity was violated, a Greenhouse–Geisser correction was applied to adjust the degrees of freedom for the repeated measures analysis. Peak force, average power at BPT, ultrasound measurements, soreness, and CPK data were analyzed using a two-factor (condition × time) fully repeated measures analysis of variance (ANOVA). Partial eta-squared values (η^2^_p_) were interpreted using the following benchmarks: small (≥ 0.01), medium (≥ 0.06), and large (≥ 0.14) (Cohen 2013). In the event of a significant F ratio, dependent *t* tests with a Bonferroni adjustment were used to examine pairwise comparison between trials for each time point. Dietary data were analyzed using a one-way ANOVA, while peak force and internal peak force at baseline in LML and SML test were analyzed using a paired-sample *t*-test. Additionally, Cohen’s d effect sizes (ES) were calculated to quantify the magnitude of differences between interactions, with thresholds defined as small > 0.2, medium > 0.6, large > 1.20, and very large > 2.0 (Hopkins et al. 2009). All statistical analyses were executed using SPSS software (version 28, IBM, USA), with a significance level set at *p* ≤ 0.05.

## Results

### Dietary logs

The participants’ average daily energy intakes during each trial were 2907.1 ± 349.0 kcal/day, 2887.2 ± 341.8 kcal/day, and 2890.8 ± 378.7 kcal/day for the LML, SML, and VAR protocols, respectively. No significant differences between the protocols were detected for the energy intake (*p* = 0.92; F = 0.007; η^2^_p_ = 0.005).

#### Exercise protocols

**Fig. 3:**
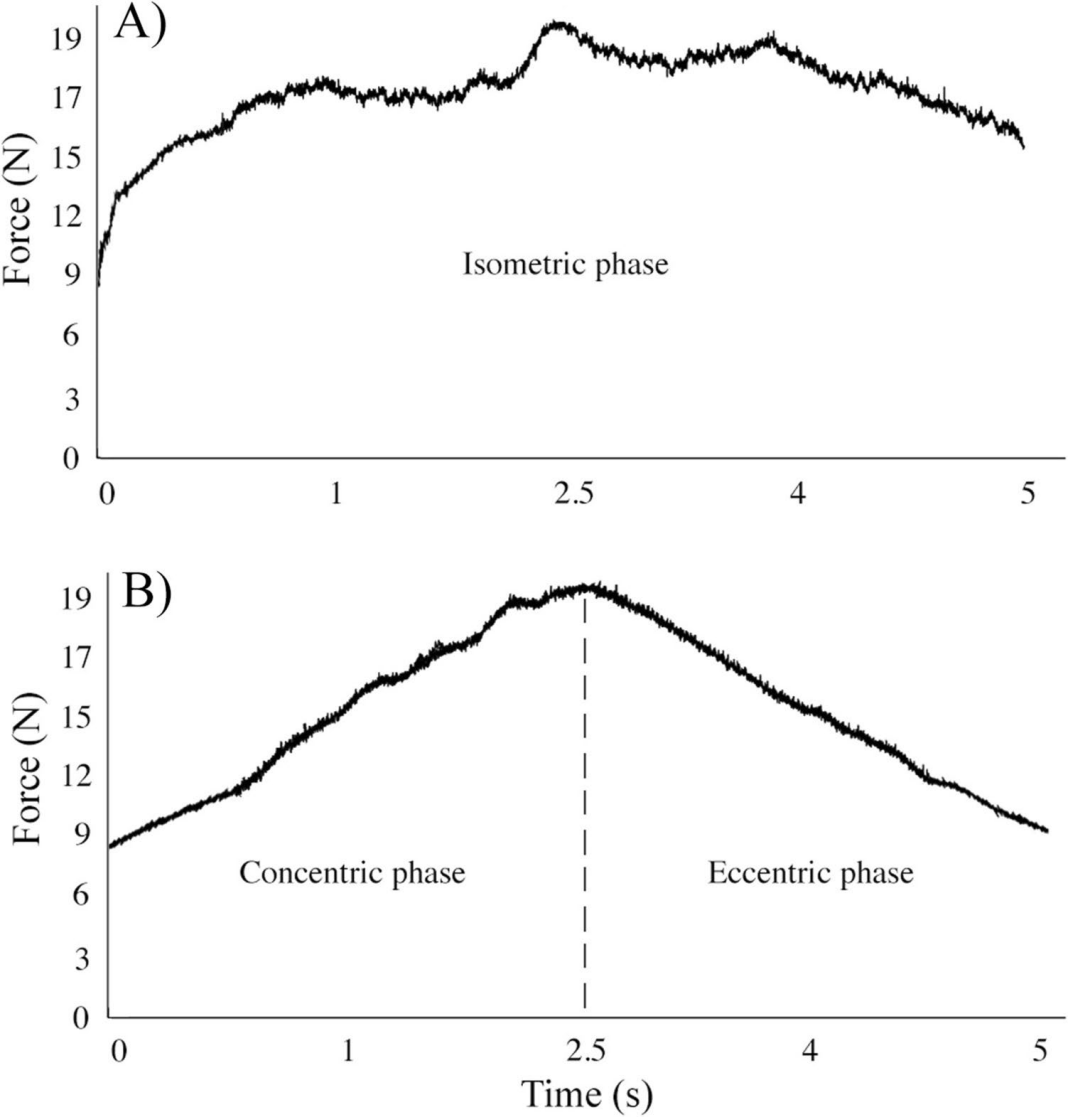
Example of a typical force–time curve obtained during an isometric contraction performed at LML or SML (**A**) and during a single repetition of VAR (**B**). These exercises mainly involved the pectoralis major muscles. *LML* long muscle length, *SML* short muscle length, *VAR* variable resistance

**Fig. 4:**
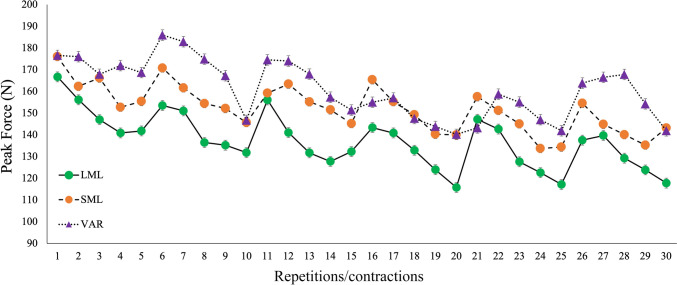
Peak force registered in each repetition/contraction of the different exercise conditions (condition average). *LML* long muscle length, *SML* short muscle length, *VAR* variable resistance

Typical examples of the force–time curve registered during an isometric contraction and during a VAR elastic band-resisted repetition are reported in Fig. 3 A, B, respectively. Figure 4 reports the average peak force registered in each contraction of LML, SML, and in each repetition of VAR protocol. The average peak force recorded during the first set (first 5 contractions/repetitions) of each exercise protocol was 150.5 ± 41.2 N, 162.5 ± 30.2 N, and 172.2 ± 29.1 N for LML, SML, and VAR protocols, respectively. In the VAR exercise protocol, adjustments of the resistance were typically performed by the investigators, following the first or the second repetition of each set (Fig. 4). The fatigue index indicated a decrease in peak force by −17%, −13% and −9% in LML, SML, and VAR protocol, respectively. No significant protocol × time interactions in the fatigue index were registered (*p* = 0.125; *F* = 2.217; η^2^_p_ = 0.122). Significant main effect of time was detected (*p* = 0.003; *F* = 14.960; η^2^_p_ = 0.599).

#### Strength and power assessments

All the data collected for the protocols are reported in Table 1 (strength, power, damage, and soreness) and Table 2 (muscle architecture). No significant differences were detected for peak force between LML and SML tests at BL (*p* = 0.258; ES = −0.34). However, the internal peak force estimated at BL in LML condition (1999.7 ± 490.9 N) was lower by 33% (*p* < 0.001; ES = −2.498) compared to the same parameter estimated in SML (2997.7 ± 604.2 N).

**Table 1:** Data relative to strength and power assessments (peak force, *BPT* bench press throw power test), muscle soreness (*VAS* visual analog scale) and muscle damage (*CPK* creatine phosphokinase) registered at the different time points following the three conditions (*LML* long muscle length, *SML* long muscle length and *VAR* variable resistance)

	Assessment	Protocol	BL	P-15 min	P-24 h	P-48 h
Strength and power assessments	LML peak force (*N*)	LML	194.3 ± 49.2	171.8 ± 32.3*	184.1 ± 34.9	190.1 ± 39.7
		SML	190.0 ± 34.6	175.3 ± 31.1*	184.1 ± 34.3	196.1 ± 32.3
		VAR	189.5 ± 30.2	170.6 ± 28.5*	186.4 ± 29.2	191.1 ± 38.8
	SML peak force (*N*)	LML	207.3 ± 44.1	196.2 ± 33.1	201.6 ± 38.3	213.0 ± 43.3
		SML	205.4 ± 38.1	199.9 ± 30.1	202.7 ± 27.8	206.2 ± 29.3
		VAR	195.0 ± 27.3	179.0 ± 28.9	200.6 ± 24.5	204.8 ± 30.5
	Average power at BPT (*W*)	LML	449.1 ± 85.9	425.4 ± 82.4*	445.5 ± 81.0	449.2 ± 78.6
		SML	448.6 ± 81.0	435.2 ± 81.4*	441.4 ± 75.6	448.5 ± 77.0
		VAR	445.4 ± 80.2	425.2 ± 63.9*	442.7 ± 65.0	453.8 ± 72.3
Soreness	VAS (mm)	LML	0.0 ± 0.0	42.3 ± 23.7*	18.4 ± 14.4*	6.6 ± 10.1*
		SML	0.0 ± 0.0	29.0 ± 19.2*	6.5 ± 10.9*	3.0 ± 6.1*
		VAR	0.0 ± 0.0	46.0 ± 22.2*	32.0 ± 25.4*	20.5 ± 23.3*
Muscle damage	CPK (U/L)	LML	284.5 ± 134.7	–	387.1 ± 159.8*	–
		SML	335.4 ± 174.1		396.7 ± 199.6*	
		VAR	286.2 ± 172.3		362.7 ± 170.7*	

“*” indicates a significant main effect of time compared to *BL* baseline

**Table 2:** Data relative to muscle architecture registered at the different time points following the three exercise protocols (*LML* long muscle length, *SML* long muscle length and *VAR* variable resistance)

	Assessment	Protocol	BL	P-15 min	P-24 h	P-48 h
Muscle architecture and muscle texture	MT (mm)	LML	2.11 ± 0.46	2.23 ± 0.38*	2.15 ± 0.41*	2.08 ± 0.41
		SML	2.12 ± 0.46	2.22 ± 0.51*	2.16 ± 0.48*	2.11 ± 0.48
		VAR	2.09 ± 0.5	2.36 ± 0.49#*	2.22 ± 0.51#*	2.07 ± 0.47
	EI (a.u)	LML	66.62 ± 11.1	69.98 ± 0.02*	67.13 ± 12.99	65.66 ± 10.20
		SML	66.53 ± 8.98	70.32 ± 9.34*	67.47 ± 9.41	67.52 ± 9.41
		VAR	65.79 ± 12.71	72.33 ± 9.78*	64.3 ± 11.47	65.24 ± 11.58
	ASM	LML	0.00439 ± 0.0006	0.00455 ± 0.0006	0.00500 ± 0.0009	0.00504 ± 0.0012
		SML	0.00469 ± 0.0009	0.00455 ± 0.0010	0.00471 ± 0.0013	0.00480 ± 0.0010
		VAR	0.00456 ± 0.0008	0.00470 ± 0.0008	0.00501 ± 0.0014	0.00516 ± 0.0018
	Entropy	LML	6.02 ± 0.16	5.96 ± 0.17	5.87 ± 0.21	5.90 ± 0.24
		SML	5.96 ± 0.18	5.97 ± 0.21	5.96 ± 0.26	5.96 ± 0.23
		VAR	5.99 ± 0.17	5.92 ± 0.16	5.90 ± 0.25	5.89 ± 0.27
	Correlation	LML	0.00261 ± 0.0007	0.00298 ± 0.0007	0.00308 ± 0.0009	0.00270 ± 0.0007
		SML	0.00266 ± 0.0006	0.00288 ± 0.0008	0.00265 ± 0.0010	0.00250 ± 0.0010
		VAR	0.00241 ± 0.0004	0.00318 ± 0.0006	0.00272 ± 0.0009	0.00311 ± 0.0010
	Contrast	LML	21.60 ± 4.54	19.12 ± 5.41	20.60 ± 5.55	19.04 ± 3.42
		SML	20.22 ± 3.42	18.62 ± 3.15	20.34 ± 6.79	19.51 ± 4.51
		VAR	19.83 ± 5.28	19.01 ± 3.35	19.62 ± 3.18	23.64 ± 3.08
	IDM	LML	0.449 ± 0.029	0.454 ± 0.034	0.468 ± 0.035	0.474 ± 0.039
		SML	0.462 ± 0.036	0.460 ± 0.040	0.462 ± 0.037	0.469 ± 0.031
		VAR	0.462 ± 0.039	0.460 ± 0.031	0.476 ± 0.038	0.459 ± 0.036

*ASM* angular second moment, *EI* echo intensity, *IDM* inverse difference moment

^“#”^ indicates a significant condition × time interaction. “*” indicates a significant difference from BL with groups combined (main effect of time)

**Fig. 5:**
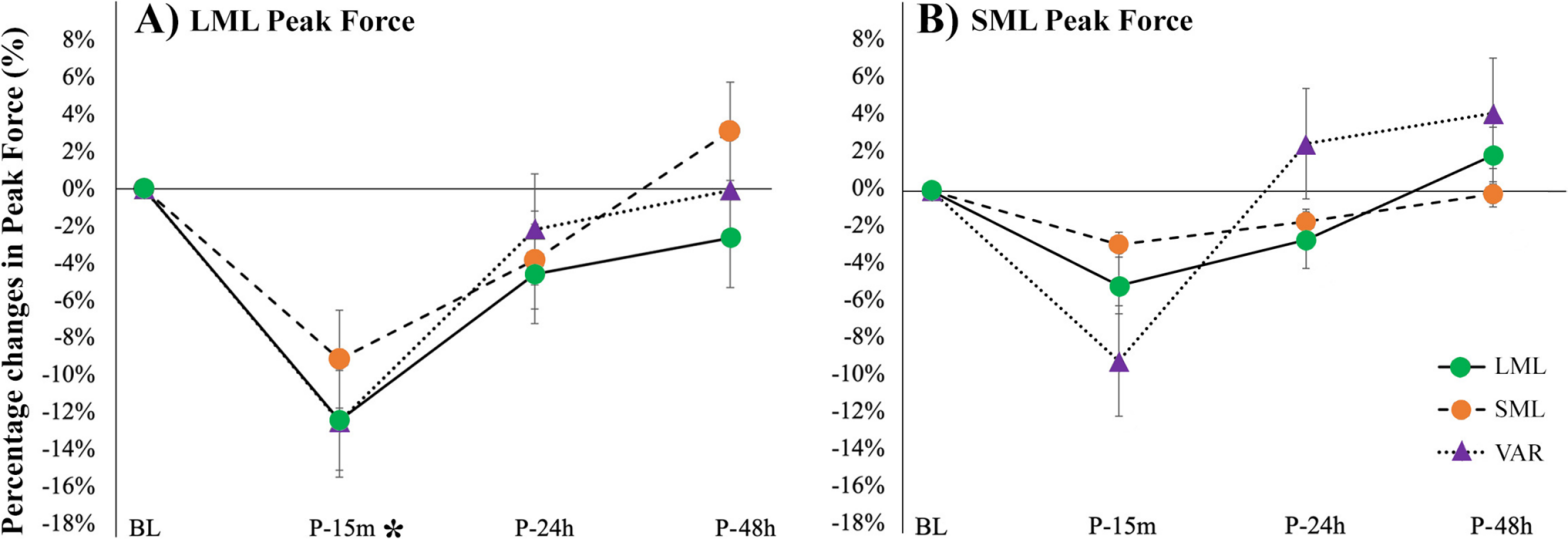
Percentage changes in LML peak force (**A**) and SML peak force (**B**) at the different time points following the three exercise protocols *LML* long muscle length, *SML* long muscle length and *VAR* variable resistance. “*” indicates a significant difference from BL with groups combined (main effect of time). Data are reported as means ± SE

**Fig. 6:**
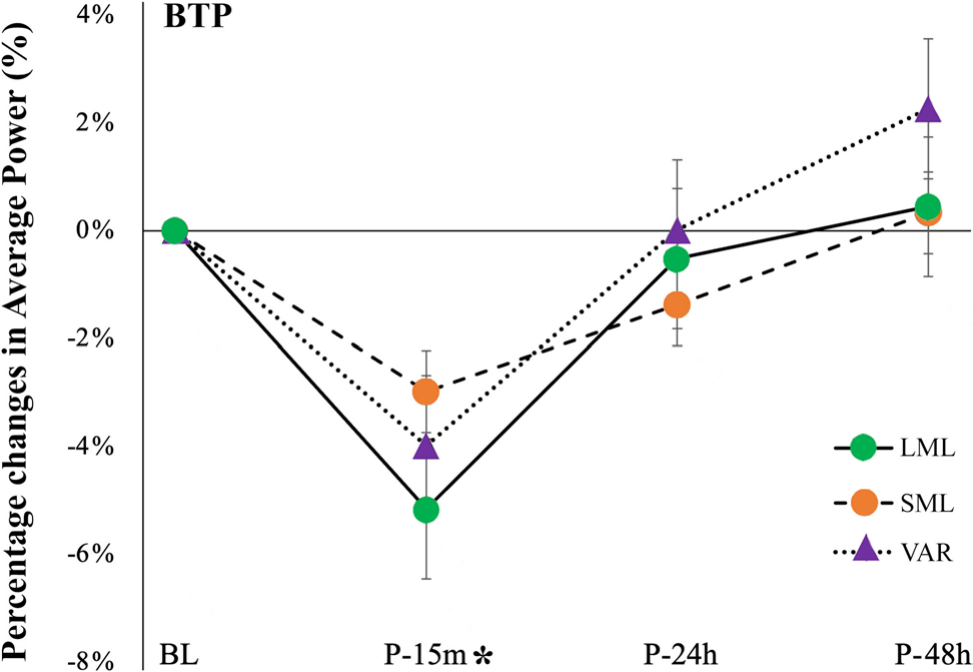
Percentage changes in average power at BPT (bench press throw power test). “*” indicates a significant difference from BL with groups combined (main effect of time). Data are reported as means ± SE. *LML* long muscle length, *SML* short muscle length, *VAR* variable resistance

In addition, no significant condition × time interactions were detected for peak force measured at LML (*p* = 0.891; *F* = 0.377; η^2^_p_ = 0.036), peak force at SML (*p* = 0.319; *F* = 1.200; η^2^_p_ = 0.107) and for average power at BPT (*p* = 0.614; *F* = 0.748; η^2^_p_ = 0.070). However, significant main effects of time were detected for peak force at LML (*p* < 0.001; *F* = 7.980; η^2^_p_ = 0.444), peak force at SML (*p* < 0.001; *F* = 7.048; η^2^_p_ = 0.413), and average power at BPT (*p* < 0.001; *F* = 19.31; η^2^_p_ = 0.659). Differences from baseline were significant at P-15 m only for peak force at LML (*p* = 0.002; ES = 0.506) and for average power at BPT (*p* < 0.001; ES = 0.235) (Figs. 5, 6).

#### Ultrasound measurements

**Fig. 7:**
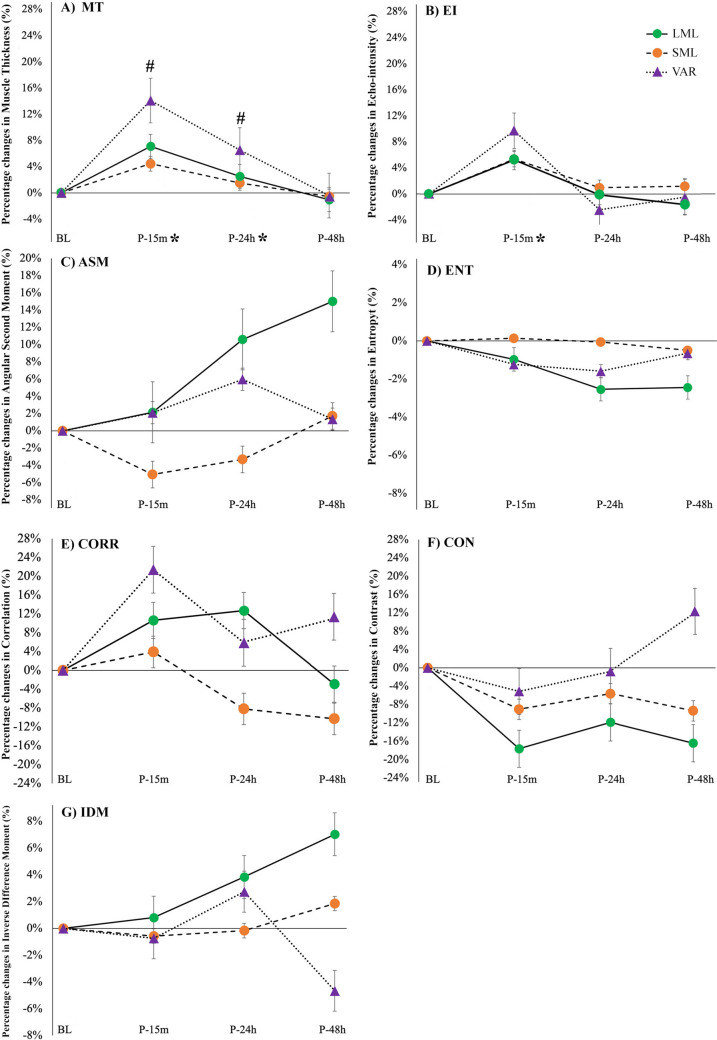
A Percentage changes of MT: **A** EI, **B** ASM, **C** ENT, **D** CORR, **E** CON and **F** IDM, **G** at the different time points of the three exercise protocols (*LML* long muscle length, *SML* short muscle length, and *VAR* variable resistance). *MT* muscle thickness, *EI* echo intensity, *ASM* angular second moment, *ENT* entropy, *CORR* correlation, *CON* contrast, *IDM* inverse difference moment. ^**“#”**^ indicates a significant condition × time interaction. “*” indicates a significant difference from BL with groups combined (main effect of time). Data are reported as means ± SE

A significant condition × time interaction was found for MT (*p* = 0.003; *F* = 3.726; η^2^_p_ = 0.271). Post hoc comparison showed significant differences between the different time points compared to BL in VAR only (*p* < 0.001; ES = 0.552 and *p* = 0.008; ES = 0.263 for P-15 m and P-24 h, respectively). A significant main effect of time (*p* < 0.001; F = 33.765; η^2^_p_ = 0.772) was also detected for MT. With the three protocols combined, MT was significantly increased at P-15 m (*p* < 0.001; ES=−0.33) and P-24 h (*p* = 0.012; ES = −0.14) compared to BL. No significant condition × time interactions were found for EI (*p* = 0.233; *F* = 1.392; η^2^_p_ = 0.122), but a main effect of time only was detected (*p* < 0.001; *F* = 9.558; η^2^_p_ = 0.489). With conditions combined, a significant increase from BL was noted at P-15 m only (*p* < 0.016; ES = −0.430). Additionally, no significant condition × time interactions were detected for angular second moment (*p* = 0.732; *F* = 0.593; η^2^_p_ = 0.165), contrast (*p* = 0.668; *F* = 0.679; η^2^_p_ = 0.185), correlation (*p* = 0.844; *F* = 0.438; η^2^_p_ = 0.127), entropy (*p* = 0.911; *F* = 0.333; η^2^_p_ = 0.100), and inverse difference moment (*p* = 0.459; *F* = 0.993; η^2^_p_ = 0.249) (Fig. 7).

#### Muscle soreness and damage

**Fig. 8:**
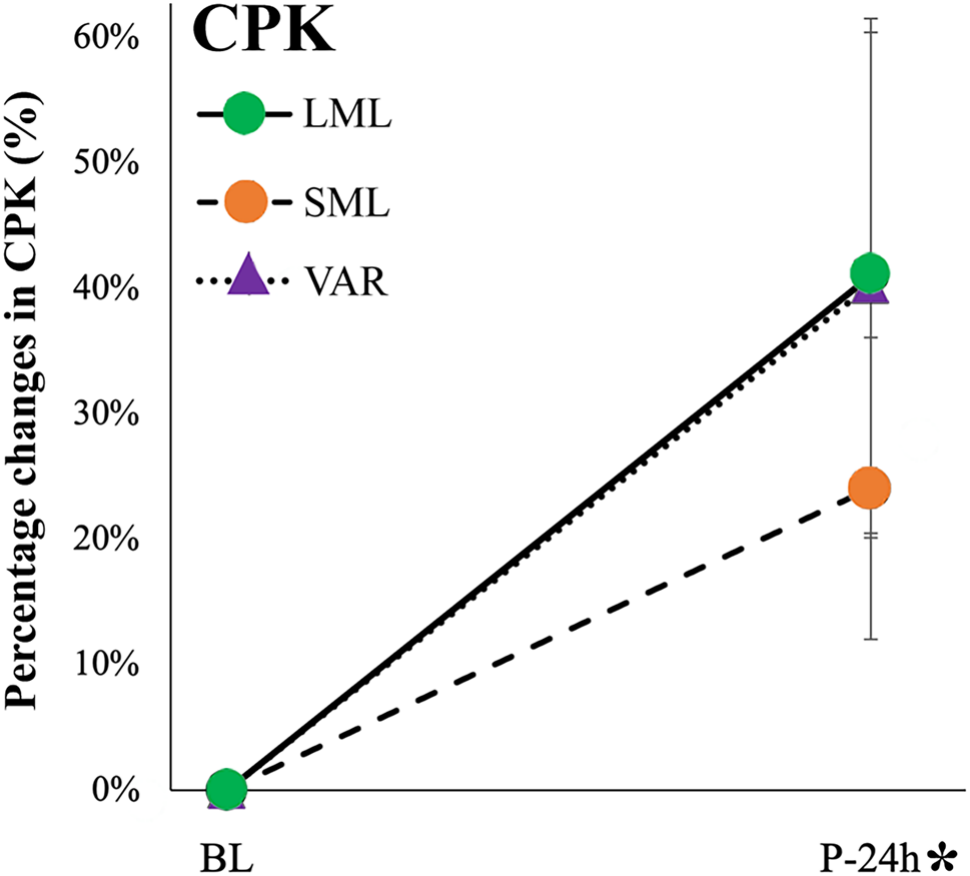
Percentage changes in CPK following the three exercise protocols (*LML* long muscle length, *SML* long muscle length and *VAR* variable resistance). *CPK* creatine phosphokinase. Data are reported as means ± SE. “*” indicates a significant difference from BL with groups combined (main effect of time)

Figure 8 shows the changes in muscle soreness and CPK at the different time points. No significant condition × time interactions were found for VAS (*p* = 0.115; *F* = 2.247; η^2^_p_ = 0.183) and CPK (*p* = 0.727; F = 0.325; η^2^_p_ = 0.035). A significant main effect of time was detected for VAS (*p* < 0.001; *F* = 48.117; η^2^_p_ = 0.828) and CPK (*p* = 0.012; *F* = 9.912; η^2^_p_ = 0.524). When the groups were combined, a significant difference with VAS BL was detected at P-15 m (*p* < 0.001; ES = −2.567), P-24 h (*p* < 0.001; ES = −1.189), and P-48 h (*p* = 0.042; ES = −0.645).

## Discussion

Results of the present study partially confirmed our research hypothesis, suggesting that the recovery response was prolonged following VAR exercise protocol compared to both LML and SML isometric protocols. However, this difference between the protocols in the recovery response was significant in MT only. In particular, this parameter indicated a 24 h delay in the recovery process following VAR compared to both isometric protocols. Longer and greater changes in muscle architecture of the pectoralis major muscle were previously reported following an eccentrically loaded bench press protocol compared to a standard high-intensity exercise regimen (Bartolomei et al. \). Thus, the longer time to recover of muscle architecture observed in the present study following VAR compared to both LML and SML may be primarily attributed to the mechanical stress associated to the eccentric component present in VAR only. In addition, the concentric phase, performed in VAR only, may have enhanced the metabolic stress typically associated to that type of muscle contraction (Paulus et al. 2019). Mechanical and metabolic stress are associated to temporary increases in muscle thickness registered following resistance exercise (Bartolomei et al. 2017; Markus et al. 2021). In addition, a transient reduction of intramuscular glycogen levels, elevated internal pressure primarily due to the accumulation of interstitial fluids and metabolites, and a temporary rise in the local blood flow, likely affect muscle texture, echo intensity and muscle thickness (Hill and Millán 2014; Markus et al. 2021). Recent studies, indeed, suggest that these physiological responses may alter the entropy (texture chaos) of the ultrasound image (Li et al. 2020) and other parameters of muscle texture (e.g., correlation). Although a trend toward an elevation of the correlation of muscle texture (+ 21.4%) was registered 15 min following the VAR protocol, no significant changes in any parameters of muscle texture were identified in the present study. The absence of maximal eccentric and isometric contractions to failure in the present study may have produced different results compared to da Matta (2018) and Li (2020), respectively.

Interestingly, no differences were noted for the drop in power following the exercise protocols. While some authors have attributed a high degree of sensitivity for exercise-induced muscle damage to upper body dynamic assessments such as the bench throw, no differences between the exercise protocols were detected in the present study. However, our results demonstrated that isometric and VAR exercises can induce muscle damage and promote increases in post-exercise circulating CPK concentrations. Despite that the absolute concentrations of CPK were slightly higher at P-24 h in LML (387.1 ± 159.8 U/L) and SML (396.7 ± 199.6 U/L) compared to VAR (362.7 ± 170.7 U/L), no significant differences in this parameter were observed between the protocols. This finding are consistent with McMahon and Onambele-Pearson, who reported comparable CPK responses across different isometric exercise protocols focusing on the leg extensor muscles (McMahon and Onambele-Pearson 2024). It is important to highlight however, that this is the first study to investigate muscle damage following VAR exercise.

Not consistent with previous investigations (Philippou et al. 2003; Allen et al. 2018; McMahon and Onambele-Pearson 2024), isometric exercise performed at LML did not result in a greater or more prolonged decline in performance compared to isometric exercise performed at SML. These studies, however, considered the elbow flexor (Allen et al. 2018; Philippou et al. 2003) or the leg extensor muscles (McMahon and Onambele-Pearson 2024), and no studies to date have been conducted on the pectoralis major muscles. The lack of differences between the isometric protocols may be partially attributed to the higher internal force calculated at SML compared to LML and to the higher structural vulnerability of the sarcomeres that typically characterizes LML (Morgan 1990). Thus, similar muscle damage and recovery responses may be produced by higher internal forces at SML or by lower internal forces at LML. Not consistently with the present study, higher internal forces at LML compared to SML were reported by some authors in some isometric exercises (e.g., leg extension) (Kubo et al. 2006; McMahon and Onambele-Pearson 2024). In these studies, a combination of the high structural vulnerability of LML and a high internal force produced more muscle damage and longer recovery responses compared to SML (Kubo et al. 2006; McMahon and Onambele-Pearson 2024). Although the exact location of LML and SML on the force–length relationship of the pectoralis major muscle was not investigated, both settings represent extreme conditions that may not maximize external forces. Some authors reported higher levels of fatigue using a VAR exercise protocol using cam system, compared to a regular resistance exercise protocol (Walker et al. 2013). The resistance provided by other mechanisms (e.g., cams or chains) may be similar to that from elastic bands; however, the latter present unique characteristics such as the lack of inertia and the tendency to increase the movement velocity during the eccentric phase (Stevenson et al. 2010). In addition, the present study was the first to quantify the force generated by elastic bands throughout the full range of motion. This strategy consented to investigate the effects of fatigue from the first to the last contractions/repetitions of each protocol. Although drops in force were registered through the exercise protocols, no differences in muscle soreness were detected between the three protocols. However, the analysis of muscle soreness may suggest a non-significant, but clinically relevant (Gallagher et al. 2001) difference between VAR and both isometric protocols at P-48 h.

A possible limitation of the present study is represented by the fact that CPK was measured at BL and 24 h following each exercise protocol only. A measurement of this parameter at P-48 h may be more representative of the amount of muscle damage that occurred in the muscles. Other limitations include a sample size of 12 participants that may not be large enough to identify small differences and interactions between the conditions. Furthermore, these results may be generalizable only to young, healthy, resistance trained men. Another possible limitation is represented by the use of the bench press throw exercise to assess the recovery response of the pectoralis major muscle following pure adduction exercise protocols. Future investigations should assess variation in the muscle texture following different volumes, intensities or specific types of contractions.

In conclusion, the present study was the first to detect muscle damage following elastic band resistance exercises and to compare the effects of upper body isometric exercises performed at long and short muscle lengths on the parameters of neuromuscular function and muscle texture of the PEC muscles. Both conditions of muscle length may result in similar muscle damages, soreness and drops in performance that can be recovered in the 24 h following each exercise protocol. Muscle thickness only showed significant longer recovery time following the VAR compared to isometric protocols.

## Data Availability

The raw data are available on request from the corresponding author.

## Abbreviations

1RM: One repetition maximum
BL: Baseline
BPT: Bench press throw power test
CPK: Creatine phosphokinase
EI: Echo intensity
LML: Long muscle length
MT: Muscle thickness
P-15min: 15 Minutes post-exercise
P-24h: 24 Hours post-exercise
P-48h: 48 Hours post-exercise
SML: Short muscle length
VAR: Variable resistance
VAS: Visual analog scale
